# Differential proteomics profiling of the ova between healthy and *Rice stripe virus*-infected female insects of *Laodelphax striatellus*

**DOI:** 10.1038/srep27216

**Published:** 2016-06-09

**Authors:** Beibei Liu, Faliang Qin, Wenwen Liu, Xifeng Wang

**Affiliations:** 1 State Key Laboratory for Biology of Plant Diseases and Insect Pests, Institute of Plant Protection, Chinese Academy of Agricultural Sciences, Beijing, China

## Abstract

*Rice stripe virus*-infected females of the small brown planthopper (SBPH, *Laodelphax striatellus*) usually lay fewer eggs with a longer hatch period, low hatchability, malformation and retarded or defective development compared with healthy females. To explore the molecular mechanism of those phenomena, we analyzed the differential proteomics profiling of the ova between viruliferous and healthy female insects using an isobaric tag for relative and absolute quantitation (iTRAQ) approach. We obtained 147 differentially accumulated proteins: 98 (66.7%) proteins increased, but 49 (33.3%) decreased in the ova of the viruliferous females. RT-qPCR was used to verify the 12 differential expressed proteins from iTRAQ, finding that trends in the transcriptional change for the 12 genes were consistent with those at the proteomic level. Differentially expressed proteins that were associated with meiosis (serine/threonine-protein phosphatase 2B and cyclin B3) and mitosis (cyclin B3 and dynein heavy chain) in viruliferous ova may contribute to low hatchability and defective or retarded development. Alterations in the abundance of proteins involved in the respiratory chain and nutrition metabolism may affect embryonic development. Our study begins to explain macroscopical developmental phenomena and explore the mechanisms by which *Rice stripe virus* impacts the development of SBPH.

The small brown planthopper (SBPH, *Laodelphax striatellus*), an important field pest, can seriously harm grain crops such as rice, not only by sucking the sap of gramineous plants, but also by transmitting several viruses, including *Rice stripe virus* (RSV), *Rice black-streaked dwarf virus* and *Maize rough dwarf virus*, which can lead to more significant yield losses after virus infection^1^. For example, rice stripe disease caused by RSV commonly causes about 20–30% losses in *japonica* rice-grown regions of China^2^. An epidemic of rice black-streaked dwarf disease affected 11.79 × 10^4^ ha in Jiangsu Province from 1991 to 2002 and then expanded into adjacent provinces such as Shandong and Henan^3^. Maize rough dwarf disease in Spain led to an average loss of 24% in commercial maize fields infected with the virus, up to 68% in areas with the highest incidence^4^. Among the three viruses, only RSV can be transmitted from the ovary into the eggs with high efficiency^5^.

RSV is transmitted by SBPH in a circulative, persistent and propagative manner and maternally from the ovary into 75% to 100% of the eggs, but it has not been detected in sperm^6^. When SBPH feeds on RSV-infected plants, RSV moves with the plant sap into the alimentary canal of the insect, infects the gut epithelial cells of the promesenteron where RSV replicates abundantly, then spreads into the adjacent epithelial cells and enteric muscle layer. It is then released into the hemolymph and, ultimately, infects the salivary glands and is released into the salivary ducts from where it can be transferred to new plants via the saliva released during feeding^5^. To infect the egg, RSV invades the nurse cell of the germarium through endocytosis mediated by a vitellogenin receptor and eventually enters the eggs^7^. These complex transmission and multiplication processes of the virus, recruiting of critical host proteins, likely influence the physiological and developmental processes of the insect^8^,^9^.

Many previous studies have shown that the infection by plant viruses impacts herbivorous vector insects in numerous ways. When Barley yellow dwarf viruses, vectored by the English grain aphid (*Sitobion avenae*), infect the aphid, the insect lives longer, produces more offspring and develops faster than the healthy insect^10^. However, the fecundity of the green rice leafhopper (*Nephotettix cincticeps*) is significantly lower after *Rice dwarf virus* infection^11^. *Tomato yellow leaf curl virus* can also shorten the life span of adult *Bemisia tabaci* and the number of eggs laid^12^. While *Tomato spotted wilt virus* infection does not alter the developmental period from egg to adult, the rate of reproduction and survival of its vector insect, Western flower thrip (*Frankliniella occidentalis*)^13^. For SBPH, RSV can invade eggs, where it can proliferate and accumulate from the antenatal stage to the 7^th^ day postpartum. Infection by RSV not only decreases the number of eggs laid per female, but also reduces the hatchability of viruliferous eggs. Microscopic observation of the eggs showed that nearly 25% of the viral-infected eggs were developmentally retarded or defective, and nearly 75% of the infected eggs developed slowly but without any abnormal morphology^7^. Moreover, the survival rate of 1^st^ and 2^nd^ instar nymphs was significantly reduced by 50% in the viruliferous insects compared with those without RSV^14^. RSV also shortens the 5^th^ instar stage and the total nymphal stage, which is thought to be in response to decreased egg production and to result in an increase in the distance that adults can migrate and thus transmit the virus^15^.

When several embryonic developmental genes of SBPH were subjected to RT-qPCR to analyze viral influence on eggs at the transcriptional level, the expression of *Ls-Dorsal*, *Ls-CPO* and other 11 embryonic developmental genes differed significantly in viruliferous eggs compared with noninfected eggs. A decrease in the transcription factor Dorsal, which initiates dorsal–ventral patterning in the *Drosophila* embryo, may lead to developmental abnormalities of eggs. Chorion peroxidase (CPO), which plays a role in forming the rigid, insoluble chorion of eggshell, is inhibited by RSV, which may cause a defect in the chorion and thus impair protection of the egg against other pathogens^7^. In an RT-qPCR analysis, *CYP307A1*, involved in the ecdysteroid pathway, and *JHAMT*, involved in the juvenile hormone pathway, were found to be upregulated and downregulated, respectively, in RSV-infected 5^th^ instars of SBPH and thus thought to contribute to the expedited development of the nymphs^15^. Although those studies have helped us to uncover the physiological and morphological influences of viruses on the vector insects, few studies have focused on proteomic changes in the host resulting from virus infection. Documenting disorders in protein accumulation in viruliferous insects can also be a powerful tool for revealing the mechanisms underlying developmental changes caused by the virus.

Many techniques such as 2D gel based technology and isobaric tag for relative and absolute quantitation (iTRAQ) can be used to identify variations in proteins under different conditions^16^. With a stable isotope labeling strategy, iTRAQ can simultaneously label and accurately quantify proteins, even low abundance proteins, from multiple samples^17^. So, iTRAQ is frequently used to explore virus-related questions^18^,^19^. In this study, we used iTRAQ to identify differentially expressed proteins in SBPH mature ova infected with RSV compared with uninfected mature ova to clarify protein changes that result from infection of RSV and to understand the interactions between RSV and *L. striatellus* more comprehensively. Study of the ova rather than the zygote can reveal the influence of RSV on SBPH from the beginning of embryonic development and exclude the interference of sperm, which cannot be infected with RSV.

## Results

### Detecting viruliferous ova from females

**Figure 1:**
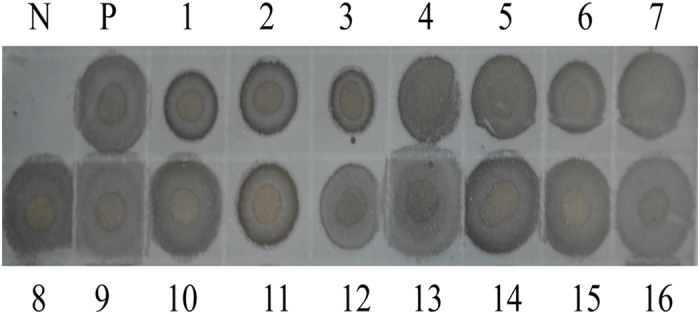
Detection of viruliferous ova excised from RSV-infected females fed on healthy plants through dot blot immunobinding assay. N: healthy SBPH; P: viruliferous SBPH; lanes 1–16: 16 nymphs borne by viruliferous female.

To ensure that viruliferous females lay a high rate of viruliferous ova, we used dot blot immunobinding assay to analyze the viruliferous rate (VR) of 16 nymphs (3^rd^ instar) from ova of viruliferous females. All 16 nymphs were viruliferous, indicating that the VR of ova laid by viruliferous females was 100%, which guaranteed the availability of the viruliferous sample for subsequent experiment and analysis (Fig. 1).

### Identification of differentially expressed proteins between viruliferous and healthy ova by iTRAQ

**Figure 2:**
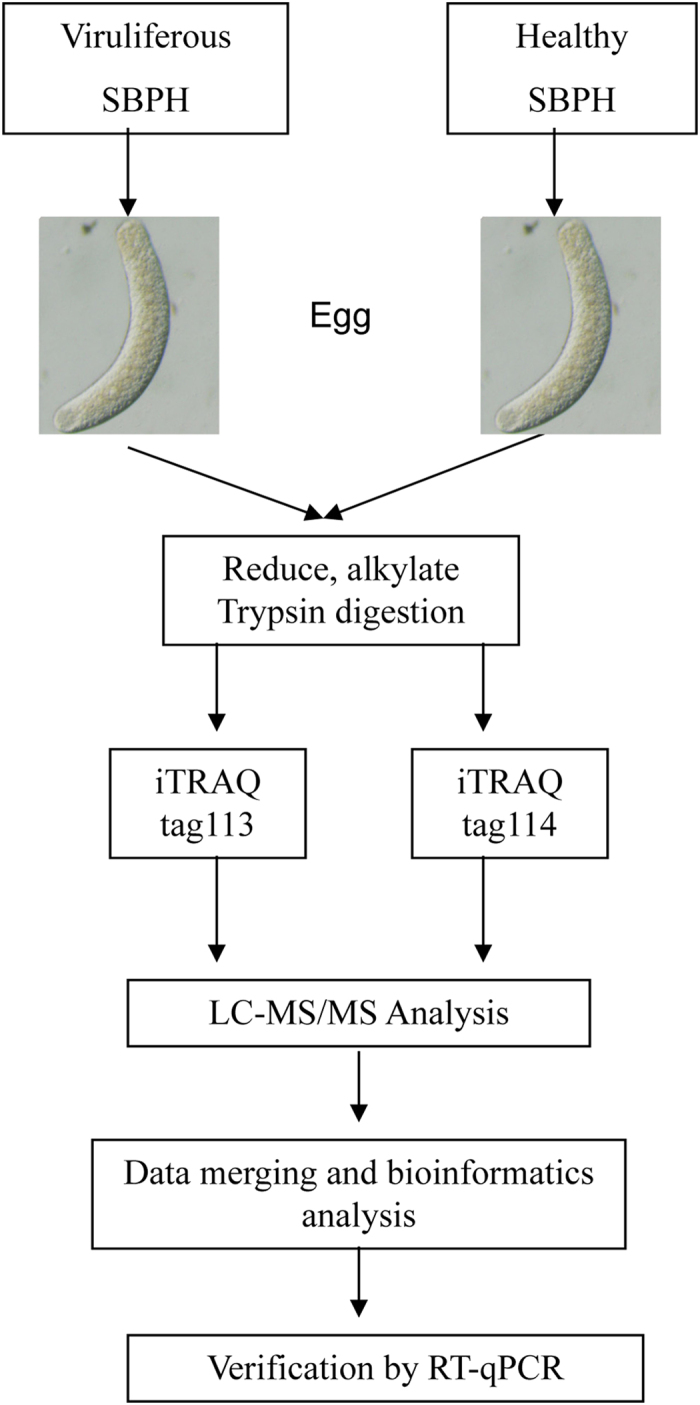
Experimental workflow. The viruliferous and healthy female SBPHs were dissected when they reached the 4^th^ peak hatching period when ova were mature, and viruliferous and healthy ova samples, respectively, were collected and lysed. Differentially expressed proteins were quantified relatively using iTRAQ labeling (tags 113 and 114, respectively) and analyzed by LC-MS/MS. At the end of the study, we conducted a general bioinformatics analysis to provide a complete list of RSV-responsive proteins in the ova and verified some proteins by RT-qPCR.

Differentially expressed proteins between RSV-infected and healthy ova were identified and quantified by 2-plex iTRAQ labeling and LC-MS/MS analysis, respectively (Fig. 2). Based on the LC-MS/MS analysis, 334 proteins were identified from the viruliferous and healthy ova. Among those proteins, 147 were differentially accumulated between the two samples (false discovery rate [FDR] < 0.01, fold changes >1.2 or <0.83): 98 (66.7%) increased and 49 (33.3%) decreased under the RSV-infection condition. Detailed information on the differentially expressed proteins, accession numbers and ratios are showed in the Table 1.

### Bioinformatics analysis

**Figure 3:**
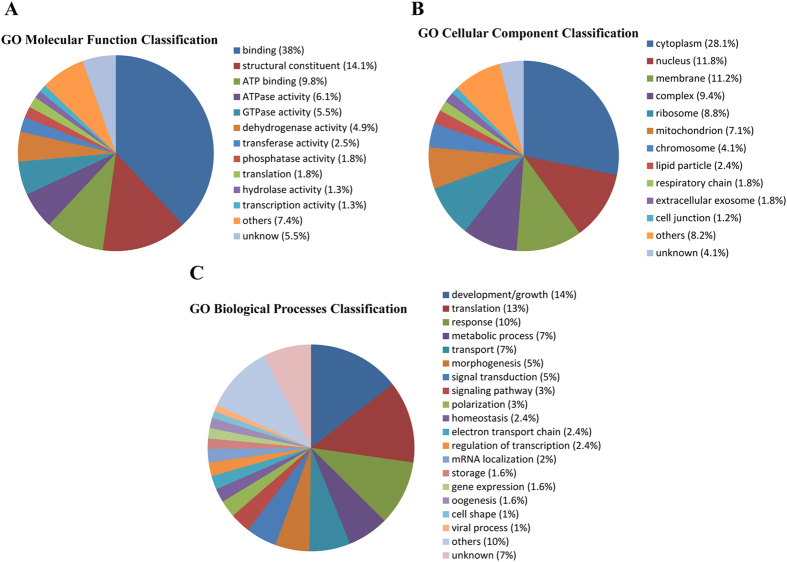
Gene ontology (GO) assignment of upregulated proteins related to molecular function, biological processes and cellular component.

**Figure 4:**
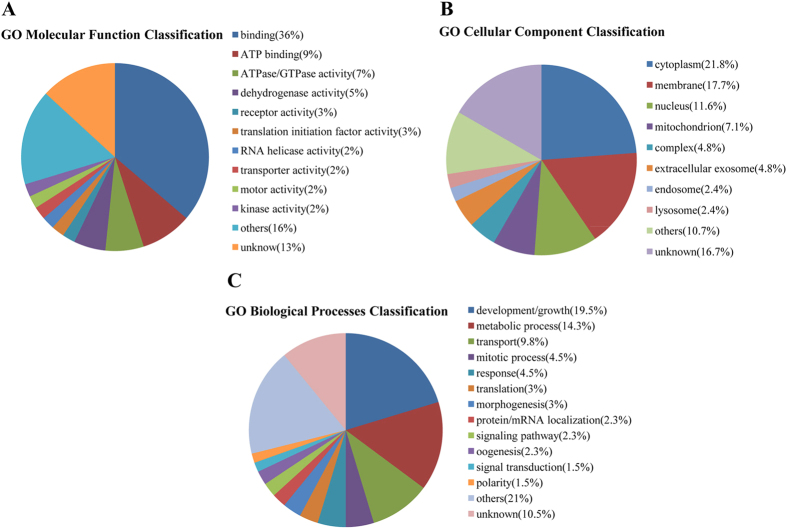
Gene ontology (GO) assignment of downregulated proteins related to molecular function, biological processes and cellular component.

**Figure 5:**
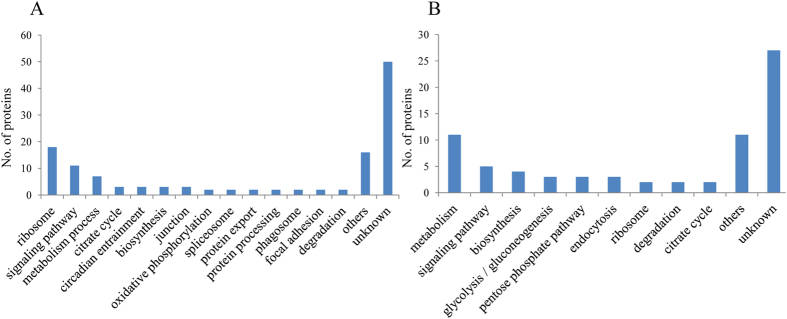
Pathway analysis of upregulated (A) and downregulated (B) proteins. The *y*-axis represents the number.

To understand the differentially accumulated proteins, the proteins were disposed by bioinformatic tools. All differentially expressed proteins were submitted to Uniprot (http://www.uniprot.org) for gene annotation, including molecular function, biological process and cellular component. For both upregulated and downregulated proteins, the main molecular functions were binding and ATP binding. According to biological process, upregulated proteins were mainly classified as development/growth, translation and response, while downregulated proteins were mainly in development/growth, metabolic process, and mitotic process. The cellular component of upregulated and downregulated proteins was mainly cytoplasm, nucleus and membrane. Detailed information can be found in Figs 3 and 4. We analyzed pathways of the differential proteins through the Kyoto Encyclopedia of Genes and Genomes (KEGG) database (http://www.kegg.jp/kegg/). In the upregulated proteins, the main pathways were related to ribosome, signaling pathway and metabolism process. Similarly, among the downregulated proteins, pathways were the dominant grouping sector for proteins involved with metabolism process, signaling pathway and biosynthesis (Fig. 5).

### Validation of the proteomics data at the RNA level by RT-qPCR

**Figure 6:**
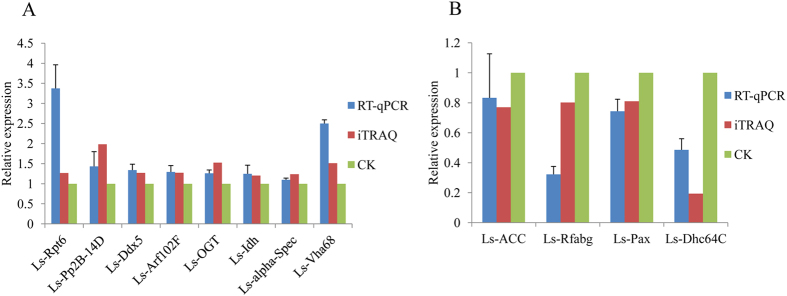
Validation of iTRAQ results through RT-qPCR of viruliferous and healthy ova samples. *β-actin* was used to normalize protein levels; mean expression levels of selected genes are denoted by the histogram bars (±SD) from triplicate repeats. Error bars represent SD. A: Eight genes (*Ls-Rpt6*, *Ls-Pp2B-14D*, *Ls-Ddx5*, *Ls-Arf102F*, *Ls-OGT*, *Ls-Idh*, *Ls-alpha-Spec* and *Ls-Vha68*) upregulated at protein and transcription levels. B: Four genes (*Ls-ACC*, *Ls-Rfabg*, *Ls-Pax* and *Ls-Dhc64C*) downregulated at the two levels. Blue and red represents the expression level of viruliferous ova using RT-qPCR and iTRAQ method respectively; gray (CK) represents that of healthy ova for negative control.

From a proteomics perspective, we found many proteins that differentially accumulated in ova infected with RSV compared with uninfected ova. To evaluate the proteomic data and correlation between mRNA transcription level and protein abundance, we performed RT-qPCR to quantify the mRNA transcript level for 12 proteins that were selected according to the proportion of their up- and downregulation and availability of the mRNA sequence from the SBPH transcriptome^20^ (Table 2). The biological processes of those proteins are mainly metabolic process (*Ls-ACC*, *Ls-Vha68*, *Ls-OGT* and *Ls-Idh*), cell cycle (*Ls-Pp2B-14D* and *Ls-Dhc64C*), response (*Ls-Rpt6*), and transport (*Ls-Arf102F* and *Ls-Dhc64C*). The trend in transcriptional variation for all the selected proteins was consistent with the proteomic changes determined in the iTRAQ analysis, suggesting that iTRAQ is a reliable way to identify and quantity the expressed differentially proteins of the SBPH (Fig. 6).

## Discussion

For nonparthenogenetic insects, such as *Drosophila melanogaster* and SBPH, the development of the mature egg is arrested during meiosis prophase I and suspended in its metabolism and cell cycle until the insect ovulates^21^. The eggs are reactivated by mechanical stimulation and hydration resulting from passage of the egg within the narrow oviducts. First, they complete meiosis, and the male and female pronuclei become integrated to form the zygote, which then undergoes rapid cleavage cycles in which the nucleus divides without cytokinesis. These processes, from the end of prophase I to the rapid cleavage cycle 13, still use the maternal mRNA and proteins; zygotic transcription does not occur until the mid-blastula transition (MBT), when the divided nucleus becomes separated by a membrane and independent cells form^22^.

Considering these processes in the context of the results of our study, we deduced that meiosis in viruliferous ova is likely to be disturbed by the decreased level of G2/mitotic-specific cyclin B3 and the increased level of serine/threonine-protein phosphatase 2B catalytic subunit 2 (Pp2B-14D). Cyclin-B3, a positive regulatory subunit of the cyclin-dependent kinase, is correlated with female fertility. In the crab ovary, increased expression of cyclin B during late vitellogenesis and final maturation of ova is considered to associated with meiotic maturation of the oocyte^23^. In addition, a mutation in cyclin B3 in *Drosophila* females leads to abnormal oogenesis, fewer eggs laid and malformed embryos^24^. In *Caenorhabditis elegans*, the loss of cyclin B3 in embryos through RNA interference (RNAi) blocks meiosis II and causes meiotic defects^25^. Pp2B-14D is a subunit of calcineurin that is necessary for meiotic progression beyond metaphase I^26^. Overexpression of a persistently active form of Pp2B-14D in a *Drosophila* female germline also causes meiotic defects^27^. Therefore, the repression of maternal cyclin-B3 and the accumulation of Pp2B-14D induced by RSV may delay the completion of meiosis and lead to defective eggs, thus explaining the low hatchability and defective development.

Our study also showed that the rapid cleavage cycles of the embryonic nucleus may be disturbed by lowered level of cell cycle-related proteins such as cyclin B3 in viruliferous ova. Cyclin B3 controls the transition from metaphase to anaphase and participates in many cell cycle events such as the timely progression of mitotis and the onset of anaphase. Knockdown of cyclin B3 in *Caenorhabditis elegans* embryo results in a longer prophase and prometaphase, and a prolonged delay in metaphase^25^. Loss of mitotic cyclins during cleavage cycles 8 and 9 in *Drosophila* as a result of RNAi causes various mitotic defects and even nuclear arrest^28^. Dynein heavy chain, cytoplasmic (Dhc64C), which was also downregulated in the viruliferous ova of SBPH, is related to spindle formation, movement of chromosomes in prometaphase and anaphase A and control of the timing of the onset of anaphase^29^. The absence of cytoplasmic dynein by RNAi leads to metaphase arrest and mitotic defects such as anaphase delay and chromosome misalignment in the *Drosophila* S2 cell line^30^. Therefore, reduced levels of cyclin B3 and Dhc64C in the viruliferous ova suggest that RSV infection may impair and arrest mitosis, which may also contribute to delayed or defective development of eggs from viruliferous females.

Previous studies have suggested that the mitochondria genes are still silenced before MBT, which means that the embryo utilizes the mitochondrial transcripts and proteins of the ova to perform respiratory-chain function^31^. But viral infection disturbs the morphology, location and respiratory chain of the mitochondria^32^. Similarly, in our study, various proteins were upregulated, e.g., many electron-transport-chain related proteins such as two components of NADH dehydrogenase (NADH dehydrogenase iron-sulfur protein 7 and 2) and cytochrome c oxidase subunit II in viruliferous ova, indicating a disorder in respiratory chain and oxidative phosphorylation compared with the healthy sample, which may impact the synthesis of ATP and influence subsequent fertilization, cleavage and embryonic development^33^. Changes in the respiratory chain caused by RSV infection may also induce the production of reactive oxygen species (ROS) beyond the level of the antioxidant system^34^. Because of the lack of mitochondrial DNA-protecting proteins and poor restoration mechanism, mitochondrial DNA (mtDNA) adjacent to the respiration chain becomes the preferred target of ROS^35^. The impairment of mtDNA may also induce the release of an apoptotic factor and result in cellular apoptosis^36^.

The yolk of the ovum contains nutrients including protein, lipid and glycogen that are transported from the nurse cells and fat body to support eventual embryonic development^37^,^38^. RSV infection induced changes in 26 proteins (17.7%) participating in metabolic processes, including protein, lipid and glycogen metabolism, which may affect nutrient utilization for embryonic development (Table 1). This alteration to nutrition may contribute to the upregulation of forkhead box protein O (FoxO), which functions to handle nutrient changes during development by regulating the insulin signaling pathway and cooperating with the cAMP pathway^39^. In *Drosophila melanogaster*, 28% of the nutrition-related genes are regulated by FoxO. Moreover, FoxO may induce a decrease in cell number by mediating insulin signaling and inhibit development through the gene *d4E-BP*^40^, which may prolong the hatch period.

Heat shock 70 kDa proteins (Hsp70) regulate translocation, the assembly and folding of proteins and inhibit the caspase-dependent apoptosis^41^. Our result showed that RSV induced the expression of Hsp70. The high level of Hsp70 is considered to improve the survival rate during hyperthermia, but may lead to the over-stimulation or -inhibition many signaling pathways related to cell multiplication, maturation and apoptosis, which will eventually influence development, growth and survival^42^,^43^.

In our study, the change in expression of some proteins may be due to aiding in replication of RSV. Without any system for protein synthesis, RSV needs to take over the ribosomal proteins (RPs) and enzymes of the host for translation of viral proteins and replication^44^. Guanine nucleotide-binding protein (RACK1), a protein that was higher in viral sample, was demonstrated to be a cellular factor that aids virus infection through an internal ribosome entry site and contributes to virus translation and replication in *Drosophila melanogaster*^45^. Through a comparative analysis of the transcriptome of SBPH, the level of two RPs was shown to increase in viruliferous insects^20^. Among the differentially expressed proteins in our study, 20 RPs were identified, and 18 (90%) accumulated to a high level in the viruliferous samples, suggesting that RPs might play key roles in viral protein synthesis for RSV duplication, indicating that RSV may proliferate and accumulate uninterruptedly in ova. Some of the enriched RPs, such as RpS23, RpL11, RPL21, RpL8, and other non-RP protein (hsp83, cyclin B3 and tubulin) also participate in the duplication of the centrosome or in the centrosome cycle^28^,^46^,^47^. The number of centrosomes has a significant impact on the number of spindle poles and accurate chromosome segregation^48^. Therefore, superabundant RPs and centrosome-related proteins for SBPH may lead to an alteration in the centrosome number, which will lead to mono- or multi-polar spindles and failure of chromosome segregation and errors in cell division^49^,^50^.

In our study, some proteins were upregulated probably to assist virus transmission in the ovum or early embryo. Transport protein Sec61 and transitional endoplasmic reticulum ATPase TER94, both upregulated in our study, participate in virus entry and infection in insects and mammals^51^. Three subunits of vacuolar ATPase (subunit E and D and catalytic subunit A) also accumulated in the viral sample; vacuolar ATPase is involved in viral entry, in releasing nucleic acid, replication and proper folding of viral proteins^52^. Meanwhile some enriched antiviral proteins were found in the differentially expressed proteins, which may inhibit the influence of RSV before or after spawning. Putative ATP-dependent RNA helicase me31b was identified as performing an antiviral function in both insect cells and adult flies^53^. Ras-related protein Rab6 regulates phagocytosis to inhibit virus infection through actin reorganization in *Drosophila melanogaster* and shrimp^54^.

In conclusion, we obtained 147 differentially expressed proteins, 98 were upregulated and 49 downregulated, in the ova between viruliferous and healthy female insects of *L. striatellus* through the iTRAQ method. Determining the variations in proteins should help us to understand the effect of RSV on SBPH ova at the proteomic level and explain phenomena such as low hatchability, developmental retardation and defects. But some protein changes induced by RSV cannot be explained well and need further experimental study. Our analysis of changes in proteins in the RSV-infected SBPH ova provides insights into the molecular mechanisms underlying RSV-induced phenomena.

## Methods

### Insect rearing and determining the infection rate of ova

Viruliferous and healthy SBPH were raised in separate glass beakers that each contained about 15 rice plants. The glass beakers were placed in an incubator at 28 °C with 16 h light/8 h dark. Rice plants are replaced with new ones every week to supply adequately nutrients. To verify whether the ova laid by viruliferous females were infected, an individual viruliferous female insect was allowed to feed in a glass beaker, and 16 offspring (3^rd^ instar nymphs) were collected and checked for RSV through a dot blot immunobinding assay using a monoclonal antibody against RSV and the method of Wang^55^.

### Protein extraction and proteinase digestion

After the female SBPH reached the 4^th^ peak hatching period^56^, we excised the ova from the ovary of the females. The samples were dissolved in moderate lysis buffer (7 M carbamide, 2 M thiocarbamide, 0.1% CHAPS) and suspended for several seconds, then broken by ultrasonication (1.2 s on, 2 s off) and then incubated at room temperature for 30 min before being centrifuged at 15,000 × *g* for 20 min at 4 °C. The supernatant was then transferred into a new tube. The Bradford method was used to measure the protein concentration^57^. After overnight digestion in 50 μL trypsin solution at 37 °C, viruliferous and healthy samples were labeled with iTRAQ reagents 113 and 114 (AB Sciex, Foster City, USA), respectively.

### 2DLC-MS/MS analysis

The labeled peptide fragments from each sample were reconstituted with mobile phase A (98% ddH_2_O, 2% acetonitrile, pH 10) and pre-separated with mobile phase B (98% acetonitrile, 2% ddH_2_O, pH 10) using RIGOL L-3000 High performance Liquid Chromatography system with an RP analytical column (Durashell-C18, 4.6 mm × 250 mm, 5 μm, 100 Å) at 0.7 mL min^−1^.Then the peptides were redissolved in 2% methyl alcohol and 0.1% formic acid and subsequently separated using a ABI-5600 system (Applied Biosystems) with an EASY-Spray column (12 cm × 75 μm, C18, 3 μm) at 350 nL min^−1^. Mobile phase A and mobile phase B were 100% H_2_O with 0.1% formic acid and 100% acetonitrile with 0.1% formic acid, respectively.

### Protein identification and quantification

Raw data were collected by Analyst QS 2.0 controlling software (AB Sciex), and Maxquant (version 1.5.2.8, Max Planck Institute of Biochemistry, Martinsried, Germany) was used to identify the proteins in a search of the protein database of Hemiptera downloaded from National Center for Biotechnology Information (NCBI, http://www.ncbi.nlm.nih.gov/). Parameters for protein identification were as follows: MS/MS tol. (FTMS) = 20 ppm, MS/MS tol (ITMS) = 0.5 Da, oxidation (M), FDR ≤ 0.01. The significantly different ratio was set at 1.2-fold: proteins were considered as upregulated if the ratio was >1.2 and downregulated if the ratio was <0.83.

### Bioinformatics analysis

Protein annotation of the identified differentially expressed proteins, including molecular function, cellular component and biological process, was performed using Uniprot (http://www.uniprot.org/)^58^ to search for comprehensive, high-quality protein functional information and mainly based on Flybase, Interpro and UniProt Knowledgebase. The pathways were analyzed using the Kyoto Encyclopedia of Genes and Genomes (KEGG) (http://www.kegg.jp/kegg/)^59^.

### Verification by real-time PCR

We used Bioedit software (version: 7.2.5.0) to create a local transcriptome of SBPH based on sequencing data assembled from SRX016333 and SRX016334 of NCBI by RunAssembly in the program Newbler (version 2.6)^60^. The 12 selected protein sequences, based on iTRAQ data, were downloaded through the accession number from the NCBI and subjected to a tBlastn similarity search against the local transcriptome. Retrieved gene sequences with an expectation value (E) less than 10^−10^ were considered to be credible and were used to design the RT-qPCR primers using the program Primer Premier Version 5.0. Viruliferous and healthy samples were triturated in TRIzol (Invitrogen) to extract total RNA. With the FastQuant RT Kit (TIANGEN), 1000 ng RNA was reverse-transcribed to synthesize cDNA. RT-qPCR was performed using the SYBR Green SuperReal PreMix (TIANGEN) with the ABI 7500 Real Time PCR thermal cycler (Applied Biosystems) and the following cycle program: 15 min at 95 °C, followed by 40 cycles of 10 s at 95 °C, 32 s at 60 °C and 72 °C for 32 s. *β-actin* was selected as a reference gene to normalize the expression level of target genes. Relative gene expression was computed using the 2^−ΔΔCT^ method^61^. The experiments were repeated 3 times independently. Healthy samples were used as a negative control. The primers used for the RT-qPCR to verify the iTRAQ result are shown in Supplementary Table S1.

## Additional Information

**How to cite this article**: Liu, B. *et al.* Differential proteomics profiling of the ova between healthy and *Rice stripe virus*-infected female insects of *Laodelphax striatellus*. *Sci. Rep.* **6**, 27216; doi: 10.1038/srep27216 (2016).

## Supplementary Material

Supplementary Information

## Figures and Tables

**Table 1:** List of differentially expressed proteins in ova of SBPH after RSV infection.

Accession number^a^	Proteins	Unique peptides^b^	Sequence coverage [%]^c^	Ratio^d^
	Translation			
662195687	eukaryotic translation initiation factor 2 subunit 3-like	1	17.4	2.484511
662197941	translationally controlled tumor protein homolog	1	6.8	2.067828
641661996	transcription elongation factor B polypeptide 2	1	6.9	1.984711
662206187	eukaryotic peptide chain release factor GTP-binding subunit ERF3A	1	4.5	1.830048
187123194	ribosomal protein L24	1	8.3	1.785167
648215847	40S ribosomal protein S5	2	8.8	1.604823
662201329	40S ribosomal protein S23	2	8.4	1.528438
641647602	60S ribosomal protein L21-like	2	12.7	1.51912
187115160	ribosomal protein S17	1	10.8	1.457962
193580101	40S ribosomal protein S9	1	16.2	1.431053
662197167	eukaryotic translation initiation factor 5A	1	5	1.415218
662199441	elongation factor Tu, mitochondrial-like	1	2.7	1.406708
662207737	60S ribosomal protein L11	1	13	1.379142
662209085	60S ribosomal protein L8	3	14.4	1.369597
662209707	60S ribosomal protein L12	1	5.5	1.350648
662184235	40S ribosomal protein S11	2	7.2	1.33406
662218739	60S ribosomal protein L4-B-like, partial	2	12.5	1.324579
187129222	ribosomal protein L34	1	6.7	1.317411
662186416	40S ribosomal protein SA	1	5.6	1.295956
662210930	60S ribosomal protein L23	1	7.1	1.270119
187129228	40S ribosomal protein S25	2	9.5	1.266763
641679542	heterogeneous nuclear ribonucleoprotein 27C-like	2	5.6	1.252255
662200191	60S ribosomal protein L32-like	1	7.5	1.221087
240849131	ribosomal protein S6	1	3.6	1.211985
641673750	ribosomal protein S28e-like	2	32.3	0.815808
662208447	eukaryotic initiation factor 4A-I-like	1	4.2	0.780624
641662274	eukaryotic translation initiation factor 3 subunit C	1	1.5	0.700217
662224988	40S ribosomal protein S8-like, partial	1	25.9	0.658957
	Metabolism			
662219845	persulfide dioxygenase ETHE1, mitochondrial	1	3.2	6.127282
328697388	2-oxoglutarate dehydrogenase, mitochondrial	1	1.4	1.652311
641677413	UDP-N-acetylglucosamine–peptide N-acetylglucosaminy-ltransferase-like	1	1.7	1.524594
641654954	V-type proton ATPase catalytic subunit A	1	8.5	1.512928
328704972	delta-1-pyrroline-5-carboxylate synthase	4	7.9	1.448002
328700405	proline dehydrogenase 1, mitochondrial	1	3.6	1.341319
328697410	DEAD-box ATP-dependent RNA helicase 20-like	2	4.2	1.32052
662214999	glutamate dehydrogenase, mitochondrial-like	2	5.3	1.302101
662210960	1,4-alpha-glucan-branching enzyme-like	1	6.2	1.27316
193700143	aldehyde dehydrogenase, mitochondrial	2	2.9	1.271559
662192943	pyruvate carboxylase, mitochondrial-like	4	3.9	1.218207
641675019	UTP–glucose-1-phosphate uridylyltransferase-like	2	4.2	1.207656
193580190	isocitrate dehydrogenase [NADP] cytoplasmic-like	2	4.9	1.20412
641659015	T-complex protein 1 subunit zeta	1	1.3	0.8241
641679894	probable pyruvate dehydrogenase E1 component subunit alpha, mitochondrial	1	2.5	0.814525
328710078	6-phosphogluconate dehydrogenase, decarboxylating	2	3.9	0.81438
662210162	probable 3-hydroxyacyl-CoA dehydrogenase B0272.3	1	3	0.793047
662201252	phosphoglycerate kinase	1	4.5	0.791029
662193985	acetyl-CoA carboxylase-like	3	2.9	0.769906
662207937	glycerol-3-phosphate dehydrogenase [NAD(+)], cytoplasmic-like	1	3.4	0.744568
328713184	very long-chain specific acyl-CoA dehydrogenase, mitochondrial	1	1.7	0.718995
193669238	translation initiation factor eIF-2B subunit delta	1	2.1	0.701824
328700025	6-phosphofructokinase	1	1.1	0.696162
641647799	histidine decarboxylase	1	2	0.689838
662205392	malate dehydrogenase, mitochondrial-like ;	2	16.9	0.67347
662195667	ribose-phosphate pyrophosphokinase 1	1	4.9	0.632195
	Electron transport			
662201650	NADH dehydrogenase [ubiquinone] iron-sulfur protein 7, mitochondrial-like	1	9.2	1.359605
671729049	cytochrome c oxidase subunit II (mitochondrion)	1	3.6	1.350648
662197766	electron transfer flavoprotein subunit beta-like	1	22.5	1.320515
662187693	NADH dehydrogenase [ubiquinone] iron-sulfur protein 2, mitochondrial	1	2.7	1.220327
	Response			
662204299	RACK1 guanine nucleotide-binding protein subunit beta-like protein	1	3.8	2.009609
662194343	guanine nucleotide-binding protein G(o) subunit alpha	1	3.7	1.984605
662191009	ras-related protein Rab6-like	2	13.9	1.652277
641673378	aldehyde dehydrogenase-like	1	2	1.569280
662216228	transitional endoplasmic reticulum ATPase TER94-like	1	3.6	1.492503
193587299	heat shock protein 70 B2-like	1	8.2	1.414212
662212992	putative ATP-dependent RNA helicase me31b	1	2.1	1.381543
328705845	dentin sialophosphoprotein	1	0.6	1.320508
662219635	heat shock protein 83-like	1	7.3	1.320508
662196678	26S protease regulatory subunit 8	4	12.4	1.267469
662199945	protein transport protein Sec61 subunit alpha	1	1.9	1.234578
662212366	ankyrin repeat domain-containing protein 17	1	0.4	1.232153
662224369	heat shock 70 kDa protein cognate 4-like, partial	1	15	1.208884
662206271	heat shock 70 kDa protein	3	5.2	1.206334
662196490	sodium/potassium-transporting ATPase subunit alpha-like, partial	3	17.4	1.224616
193652521	ATP-dependent RNA helicase WM6	1	2.4	0.696162
	Cell cycle			
662198987	serine/threonine-protein phosphatase 2B catalytic subunit 2-like	1	15.2	1.984675
662183545	titin-like	1	0.2	1.585952
662198211	cofilin/actin-depolymerizing factor homolog	2	16.5	0.821183
187179329	twinstar	1	4.7	0.79105
662201759	G2/mitotic-specific cyclin-B3-like	1	1.9	0.61666
662211502	AP-2 complex subunit alpha	1	1.4	0.493967
662185744	dynein heavy chain, cytoplasmic-like	1	0.2	0.194088
	Transport			
662197932	ras-related protein Rab-2A	1	9.4	1.984636
662183037	plasma membrane calcium-transporting ATPase 3-like	1	1.7	1.685486
326319966	V-type proton ATPase subunit D	1	2.6	1.486314
193634236	innexin inx2	1	2.5	1.486244
328711155	clathrin heavy chain	2	1.9	1.439576
662224735	V-type proton ATPase subunit E-like	1	5.8	1.358345
662218609	ADP-ribosylation factor 2-like, partial	1	8.4	1.27316
662187312	fatty acid-binding protein, muscle	1	6.8	1.237659
187121188	bicaudal	1	6.6	0.815664
662190099	apolipophorins-like	1	0.3	0.802221
641657530	glutamate receptor ionotropic, kainate 1	1	2	0.79105
662206445	ras-related protein Rab-7a	1	5.2	0.696162
641666000	ATP-binding cassette sub-family E member 1	1	1.5	0.60393
662213752	vacuolar protein sorting-associated protein 29	1	5.5	0.55669
	Transcription regulation			
662211365	forkhead box protein O-like, partial	1	3.4	2.099884
641667108	segmentation protein Runt-like	1	2.1	1.364041
662221075	histone H4	1	50.5	1.344955
328717963	probable ATP-dependent RNA helicase DDX5	1	4	1.26917
	Signal transduction			
662218361	spectrin beta chain, erythrocytic-like, partial	1	3.8	2.484511
240848699	troponin C-like	1	9.3	2.166203
648215987	FK506-binding protein 1 precursor	1	8.1	1.984675
641649210	calcium/calmodulin-dependent 3,5-cyclic nucleotide phosphodiesterase 1C-like	1	1	1.585871
662185726	ras-related protein Rab-8B-like	1	8.3	1.527754
662191522	ralA-binding protein 1-like	1	1.6	0.592953
	Others			
641667476	dystonin	1	0.2	5.332768
193618005	T-complex protein 1 subunit gamma-like	1	2	2.317678
641657060	trichohyalin-like	1	2.9	2.127308
662221462	zinc finger CCCH domain-containing protein 13-like, partial	1	1.4	2.00459
641654607	uncharacterized protein LOC103308150	1	5.1	1.984605
662206904	uncharacterized protein LOC103513857, partial	1	2.2	1.874966
641664858	uncharacterized protein LOC100571486	1	1.5	1.805057
641679348	zinc finger MYM-type protein 1-like	1	1.1	1.65225
237874213	Obg-like ATPase 1	1	2.5	1.616551
662198825	uncharacterized protein LOC103509702	1	1.5	1.478131
641659614	uncharacterized protein LOC103308837	1	0.7	1.457034
641658025	uncharacterized protein LOC100573999	1	2.1	1.422523
662203311	uncharacterized protein LOC103512009	1	2.1	1.409739
662199313	T-complex protein 1 subunit theta	2	4.7	1.355449
662224929	troponin T-like	2	5.7	1.29113
298676439	tubulin beta-1 chain	7	23.5	1.286363
641651626	tubulin beta chain-like	1	7.7	1.280335
328699232	spectrin beta chain	4	1.7	1.248244
641678036	spectrin alpha chain	2	4.2	1.238482
328708622	muscle LIM protein Mlp84B-like	2	4.8	1.231617
328709476	multiple inositol polyphosphate phosphatase 1-like	1	1.5	1.229761
328703083	alpha-actinin, sarcomeric	3	10.9	1.222747
662217109	uncharacterized protein LOC103519217	1	1.3	1.200012
662199835	neural-cadherin-like	1	6.7	0.8241
328715019	transketolase-like protein 2	1	2.1	0.819711
662194096	paxillin	1	4.8	0.810566
662187362	GTP-binding protein 1-like	1	4.6	0.803458
662199465	lisH domain and HEAT repeat-containing protein KIAA1468 homolog	1	1.2	0.784215
328702659	uncharacterized protein LOC100569797	1	1.8	0.777457
328721582	netrin-1-like	1	5.4	0.776104
662204082	myosin heavy chain, muscle-like	3	12.2	0.77215
662198855	immunoglobulin superfamily containing leucine-rich repeat protein 2-like	1	4.3	0.768863
641646988	26S proteasome non-ATPase regulatory subunit 10-like	1	8.9	0.738145
648216270	uncharacterized protein LOC100168138	1	5.9	0.706124
641673054	uncharacterized protein LOC103310269	1	2.9	0.702618
662218667	uncharacterized protein LOC103520057	1	0.8	0.648129
662197467	putative uncharacterized protein DDB_G0282133	1	0.6	0.635234
641668388	microtubule-associated serine/threonine-protein kinase 3	1	0.7	0.55669
662219363	uncharacterized protein MAL13P1.304-like, partial	1	1.1	0.542914
662189113	ubiquitin-like modifier-activating enzyme 1, partial	1	1.4	0.449017
662208914	uncharacterized protein LOC103514896	1	1.5	0.329166
662188243	uncharacterized protein DDB_G0284459-like	1	1.7	0.003

^a^Protein accession number from NCBI.

^b^Number of unique peptides identified for each protein.

^c^Percentage sequence coverage of identified proteins.

^d^Ratios of RSV-infected/mock-infected proteins.

**Table 2:** List of genes selected for RT-qPCR assay.

Accession number	Proteins	Genes	Ratio	Annotation
662196678	26S protease regulatory subunit 8	*Ls-Rpt6*	1.267469	response to DNA damage stimulus; proteasomal protein catabolic process
662198987	serine/threonine-protein phosphatase 2B catalytic subunit 2-like	*Ls-Pp2B-14D*	1.984675	meiotic division
328717963	probable ATP-dependent RNA helicase DDX5	*Ls-Ddx5*	1.26917	regulation of pre-mRNA splicing; transcriptional coactivator
662218609	ADP-ribosylation factor 2-like, partial	*Ls-Arf102F*	1.27316	protein transport
641677413	UDP-N-acetylglucosamine--peptide N-acetylglucosa- minyltransferase-like	*Ls-OGT*	1.524594	energy/glycogen metabolism
193580190	isocitrate dehydrogenase [NADP] cytoplasmic-like	*Ls-Idh*	1.20412	tricarboxylic acid cycle
641678036	spectrin alpha chain	*Ls-alpha-Spec*	1.238482	constituent of the cytoskeletal network; maintenance of cell shape
641654954	V-type proton ATPase catalytic subunit A	*Ls-Vha68*	1.512928	ATP metabolic process
662193985	acetyl-CoA carboxylase-like	*Ls-ACC*	0.769906	fatty acid metabolism
662190099	apolipophorins-like	*Ls-Rfabg*	0.802221	transport
662194096	paxillin	*Ls-Pax*	0.810566	cytoskeletal protein
662185744	dynein heavy chain, cytoplasmic-like	*Ls-Dhc64C*	0.194088	mitotic nuclear division; transport
