# Supplementary material for: Differential proteomics profiling of the ova between healthy and Rice stripe virus-infected female insects of Laodelphax striatellus
Source: Sci Rep. 2016 Jun 9;6:27216. doi: 10.1038/srep27216 (PMC4899684; doi:10.1038/srep27216)
Supplement: Supplementary Information [file srep27216-s1.pdf]

Supporting Information for

Differential proteomics profiling of the ova between healthy and *Rice stripe virus*-infected female insects of *Laodelphax striatellus*

Beibei Liu, Faliang Qin, Wenwen Liu, Xifeng Wang*

State Key Laboratory for Biology of Plant Diseases and Insect Pests, *Institute of Plant Protection*, *Chinese Academy of Agricultural Sciences*, *Beijing*, *China*

*Corresponding author: Email: xfwang@ippcaas.cn

Supplementary Table S1: Primers used in the RT-qPCR

**Gene name**	**Primer sequence (5′ to 3′)**
Ls-Rpt6-F	GCGCCGTCGATAATCTTCAT
Ls-Rpt6-R	CGGGTCCAGAATGTCTATGC
Ls-Pp2B-14D-F	TCTCGGGTGGCAAGACTTCA
Ls-Pp2B-14D-R	CGGCATTCGCTCGTTGATGG
Ls-Ddx5-F	CAAATCAGGACAGGCAAGAG
Ls-Ddx5-R	TTCATCCAAGACGAGGTAGG
Ls-Arf102F-F	AGACCACTCTGGCGGCATTA
Ls-Arf102F-R	TTGGCGAATATCAGCAGGAC
Ls-OGT-F	GGTTACGGAGGCAGAAGAAT
Ls-OGT-R	TTGAGGTAGAGTCGAGTGGC
Ls-Idh-F	CAACCAACCCTATTGCTTCC
Ls-Idh-R	CACTCCACGCATGACTCTTC
Ls-alpha-Spec-F	TTCCGTGACCTCATCTCCTG
Ls-alpha-Spec-R	ATCTCGCCCTTGTGCTCTTG
Ls-Vha68-F	CACTGGAAGTCGCCAAGGTC
Ls-Vha68-R	TGCCGAGCCATGTCGTAGAA
Ls-ACC-F	CCAACAGTGGTGCCAGAGTC
Ls-ACC-R	CGTCGATCAGAATAGCCTTG
Ls-Rfabg-F	CCATCAGACCAAAGAGGAGCAT
Ls-Rfabg-R	TACCAGCCGACTTCAGAGCC
Ls-Pax-F	GGCAGTCTACAGGCAGACAT
Ls-Pax-R	GTCGCGCTCAAAGAAGTTGC
Ls-Dhc64C-F	GAACGGAACAAGACCAAGGA
Ls-Dhc64C-R	TTGACAATGCAGTCCCACAG
